# Magnetic resonance guided adaptive stereotactic body radiotherapy for lung tumors in ultracentral location: the MAGELLAN trial (ARO 2021-3)

**DOI:** 10.1186/s13014-022-02070-x

**Published:** 2022-05-25

**Authors:** Sebastian Regnery, Jonas Ristau, Fabian Weykamp, Philipp Hoegen, Simon David Sprengel, Katharina Maria Paul, Carolin Buchele, Sebastian Klüter, Carolin Rippke, Claudia Katharina Renkamp, Moritz Pohl, Jan Meis, Thomas Welzel, Sebastian Adeberg, Stefan Alexander Koerber, Jürgen Debus, Juliane Hörner-Rieber

**Affiliations:** 1 Department of Radiation Oncology, University Hospital Heidelberg, Im Neuenheimer Feld 400, 69120 Heidelberg, Germany; 2 National Center for Radiation Oncology (NCRO), Heidelberg Institute for Radiation Oncology (HIRO), Im Neuenheimer Feld 400, 69120 Heidelberg, Germany; 3 Heidelberg Ion-Beam Therapy Center (HIT), Department of Radiation Oncology, University Hospital Heidelberg, Heidelberg, Germany; 4 National Center for Tumor Diseases (NCT), Heidelberg, Germany; 5 Clinical Cooperation Unit Radiation Oncology, German Cancer Research Center (DKFZ), Heidelberg, Germany; 6 Institute of Medical Biometry, University Hospital Heidelberg, Im Neuenheimer Feld 130.3, 69120 Heidelberg, Germany

**Keywords:** SBRT, IGRT, Safety, Dose-escalation, Phase 1, MR-guided radiotherapy

## Abstract

**Background:**

Stereotactic Body Radiotherapy (SBRT) is a standard treatment for inoperable primary and secondary lung tumors. In case of ultracentral tumor location, defined as tumor contact with vulnerable mediastinal structures such as the proximal bronchial tree (PBT) or esophagus, SBRT is associated with an increased risk for severe complications. Magnetic resonance (MR)-guided SBRT can mitigate this risk based on gated dose delivery and daily plan adaptation. The MAGELLAN trial aims to find the maximum tolerated dose (MTD) of MR-guided SBRT of ultracentral lung tumors (ULT).

**Patients and methods:**

MAGELLAN is a prospective phase I dose escalation trial. A maximum of 38 patients with primary and secondary ULT with a tumor size ≤ 5 cm will be enrolled. Ultracentral location is defined as an overlap of the planning target volume (PTV) with the PBT or esophagus. Patients are treated at a 0.35 Tesla MR-linac (MRIdian® Linac, ViewRay Inc. ) employing a gating strategy and daily plan adaptation. Dose escalation starts at 10 × 5.5 Gy (biologically effective dose BED_3/10_: 155.83 Gy/85.25 Gy), may proceed up to 10 × 6.5 Gy (BED_3/10_: 205.83 Gy/107.25 Gy) and is guided by a customized time-to-event continual reassessment method (TITE CRM) with backup element, which alternately assigns patients to dose escalation and backup cohorts.

**Discussion:**

The results of the MAGELLAN trial will guide further research and clinical implementation of MR-guided SBRT as ablative treatment of ULT. Moreover, the combination of MR-guided radiotherapy with TITE-CRM including a backup element may serve as blueprint for future radiation dose escalation studies in critical locations.

***Trial Registration*:**

Registered at ClinicalTrials.gov: NCT04925583 on 14th June 2021.

## Background

Stereotactic body radiotherapy (SBRT) is a long-standing standard therapy in patients with inoperable early-stage non-small cell lung cancer (NSCLC) and offers high local control of pulmonary oligometastases [1–3]. The primary determinant of local tumor control is the application of ablative biologically effective doses (α/β ratio = 10 for tumor cells, BED_10_) ≥ 100 Gy [2]. Although toxicity is low after ablative SBRT of peripheral lung tumors (e.g. 3 × 15 Gy, BED_10_ = 112.5 Gy), the risk for severe complications increases with proximity to the proximal bronchial tree (PBT) [4]. Accordingly, ablative SBRT of ultracentral lung tumors (ULT), whose gross tumor volume (GTV) or planning target volume (PTV) overlaps with the PBT or esophagus, seems to carry the highest risk for severe complications [4–6]. Only recently, results from the prospective HILUS trial confirmed this high risk: 15% of patients who received 8 × 7 Gy (BED_10_ = 95.2 Gy) to a lung tumor ≤ 1 cm from the PBT experienced a treatment-related death [6]. Therefore, many current clinical approaches use low-dose fractionation schemes that reliably reduce the risk for complications while simultaneously decreasing local tumor control (e.g. 10 × 5 Gy, BED_10_ = 75 Gy) [7].

Magnetic resonance (MR)-guided radiotherapy is an emerging technique that allows MR-imaging (MRI) before and during each treatment fraction. Consequently, the initial radiotherapy plan may be adapted based on daily MRI, thus correcting for interfractional changes in patient anatomy [8]. Moreover, gated dose delivery becomes possible, which obviates the need for large safety margins that encompass the whole tumor motion during breathing in current CT-based approaches [9, 10]. Hence, MR-guided SBRT (MRgSBRT) can correct for both intra- and interfractional motion of target volumes and organs at risk (OAR), which offers a great opportunity to precisely ablate the target while protecting surrounding OAR. Recently, we analyzed the first patients that received pulmonary MRgSBRT at our institution. Our findings support the clinical feasibility of this new technique and indeed suggest a high potential to spare OAR close to the irradiated lung tumor [8]. Therefore, MR-guided SBRT can offer a wider therapeutic ratio in the treatment of ULT. The MAGELLAN trial (ClinicalTrials.gov: NCT04925583) is a prospective phase I dose escalation trial which aims to find the maximum tolerated dose (MTD) of MR-guided SBRT of ULT.

## Patients and methods

### Objectives and endpoints

The primary objective is to estimate the MTD of MRgSBRT of ULT, defined by a dose-limiting toxicity (DLT) rate of 35%. DLT is the corresponding binary primary endpoint and encompasses pre-specified pulmonary, esophageal, cardiac or neurological complications ≥ grade 3 within 12 months of MRgSBRT based on the common terminology criteria for adverse events (CTCAE) in version 5.

Secondary objectives include description of tumor control, patient survival, patient-reported outcomes and longitudinal cardiopulmonary function. Translational objectives encompass identification of imaging biomarkers of pulmonary toxicity and tumor response from multiparametric thoracic MRI (1.5 T, T1-/T2-/diffusion-weighted) before and after treatment. Moreover, changes in serum cytokines and immunophenotypes of peripheral blood mononucleated cells (PBMC) are explored to detect early biomarkers of pulmonary toxicity and tumor response.

### Patient selection

Adult patients with primary and secondary ULT ≤ 5 cm in largest diameter and indication for SBRT according to an interdisciplinary tumor conference are eligible. Ultracentral location is defined as overlap of the PTV with the PBT (defined acc. to RTOG 0813 [11]) or esophagus. Furthermore, a Karnofsky Performace Score ≥ 70% and the ability to adequately participate in an MR-guided SBRT session are required.

### Radiotherapy

MR-guided SBRT is delivered at a MRIdian Linac® system (6 MV linear accelerator, 0.35 T MR scanner, ViewRay Inc.; Oakwood, USA) as described previously [8]. Briefly, SBRT is delivered as step-and-shoot intensity-modulated RT (IMRT) using a coplanar beam set. Before each fraction, 3D MRI is performed and the contours of the target volume and surrounding OAR are edited. Thus, the initial RT plan can be recalculated on the current anatomy and can be adapted in case of planning objective violations (e.g. insufficient PTV coverage, violation of OAR constraints). During radiotherapy, cineMRI is continuously performed to track target motion, which allows for gated dose delivery. Four different dose levels may be applied as 10-fraction schemes on successive weekdays (Table 1). The GTV is expanded by 2 mm while respecting borders of adjacent organs to create a clinical target volume (CTV). Subsequently, the CTV is expanded by 3 mm to create the PTV. The aim is a 95% coverage of the PTV by the prescribed dose with a dose maximum of 125%. Importantly, OAR constraints (Table 2) are given priority over PTV coverage. If PTV coverage aim and OAR constraints collide, PTV coverage is reduced as much as necessary to comply with OAR constraints**.**

**Table 1:** Employed dose levels

	Dose levels			
	Level 0	Level 1 (Start)	Level 2	Level 3
Single dose (Gy)	5.0	5.5	6.0	6.5
Fractions	10	10	10	10
Total dose (Gy)	50	55	60	65
BED (α/β ratio = 10, tumor cells) [Gy]	75	85.25	96	107.25
BED (α/β ratio = 3, normal tissue cells) [Gy]	133.3	155.83	180.0	205.83

*BED* biologically effective dose, *Gy* Gray

**Table 2:** Recommended dose constraints

Dose constraints		
Organ at risk	Volume	Maximum dose
Proximal bronchial tree	0.33 cm^3^	< 63.0 Gy
		< 105% prescribed dose
Non-GTV lung	1500 cm^3^	< 15.5 Gy
	1000 cm^3^	< 16.5 Gy
	< 10%	≥ 20 Gy (V_20Gy_)
Esophagus	0.5 cm^3^	< 43.5 Gy
Stomach and intestines	0.5 cm^3^	< 43.5 Gy
Heart	0.5 cm^3^	< 66.0 Gy
Aorta and major vessels	0.5 cm^3^	< 70.0 Gy
Spinal cord	0.1 cm^3^	< 35.0 Gy
Brachial plexus	0.1 cm^3^	< 39.0 Gy

*GTV* gross tumor volume, *Gy* Gray

### Dose escalation

Dose escalation will start at 10 × 5.5 Gy and will be guided by a time-to-event continual reassessment method (TITE-CRM). The TITE-CRM models the relationship between the radiation dose level and DLT rate. This model is repeatedly updated with the prospectively observed toxicity data and weighs patients according to their actual time under observation. Thus, TITE-CRM can estimate the expected toxicity on each dose level based on all hitherto available data and recommend the optimum dose level. This enables data-based patient accrual in a continuous manner [12]. Dose escalation will be performed in cohorts of 3 patients with an individual observation time for DLTs of 12 months. After a cumulative observation time ≥ 18 months (individual ≥ 3 months) in the current cohort, the TITE-CRM model is updated to recommend the dose level for the next cohort. Hence, it is possible that the next cohort is either treated at a higher (escalation) or lower (de-escalation) dose level, depending on hitherto observations. Additionally, the TITE-CRM in this trial features a backup element [13], which allows inclusion of up to six patients on the dose level below the current recommendation during the cumulative observation. Hence, patient accrual will be truly continuous. The trial will stop according to predefined stopping criteria (see below).

Additional safety rules are as follows:

- All patients in the first cohort must be observed for 12 months before dose escalation starts
- Dose escalation may not skip dose levels on the way up
- Application of an escalation with overdose control (EWOC) scheme, which does not allow the application of dose levels with potentially excessive DLT rates according to the TITE-CRM model

**Fig. 1:**
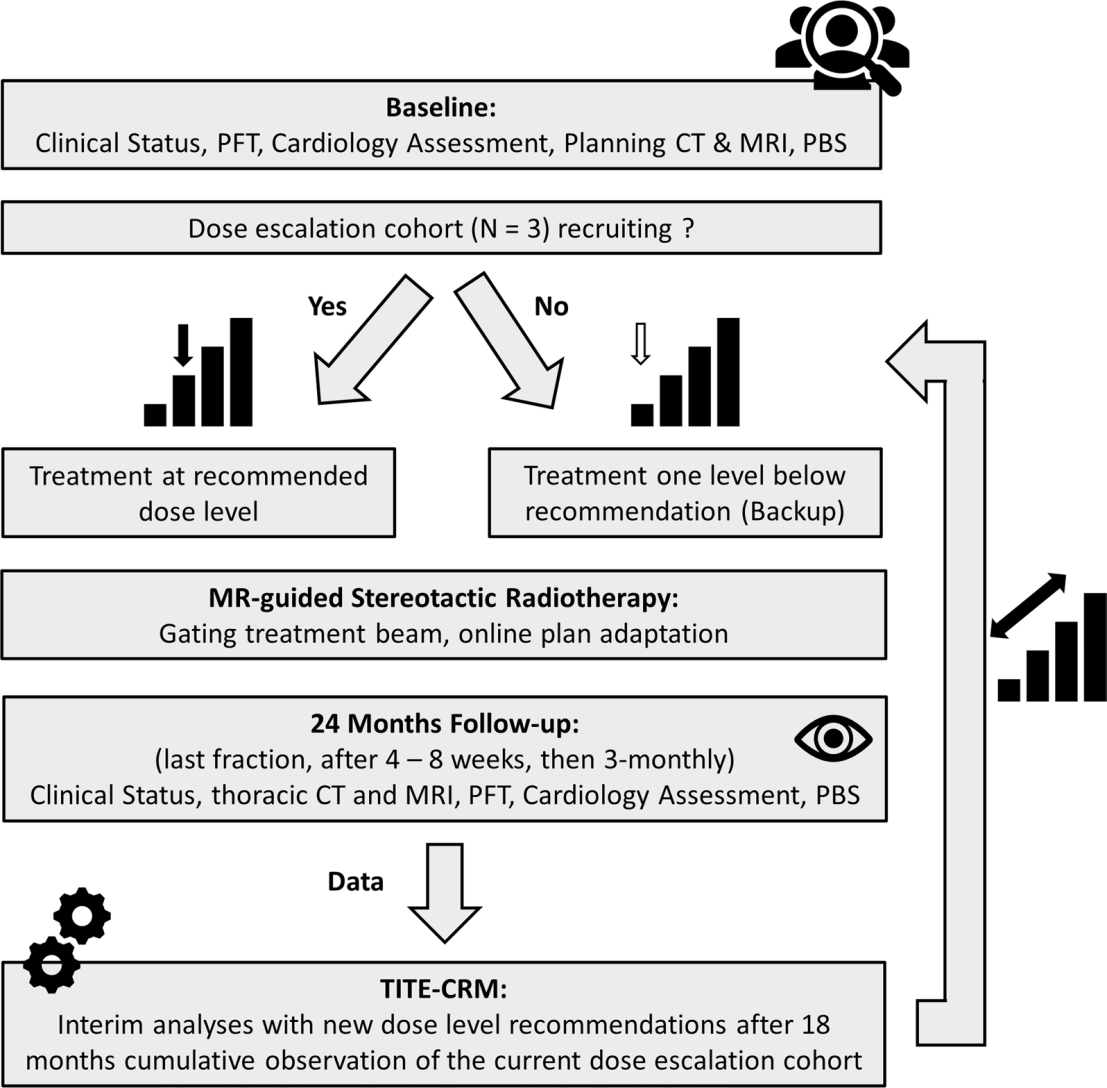
Study design. *PFT* Pulmonary Function Test, *CT* Computed Tomography, *MRI* Magnetic Resonance Imaging, *PBS* Peripheral Blood Sample, *TITE-CRM* time-to-event continual reassessment method

Figure 1 gives an overview of the study workflow.

### Statistical design

TITE-CRM is based on a Bayesian two-parametric logistic regression model [12]. In Bayesian statistics, the posterior probabilities of a set of parameters, which in this case lead to the DLT rate, are estimated given the observed data. The next dose recommendation is made for the dose level with the highest posterior probability of a DLT rate within the target interval (0.25, 0.35] and thus being closest to the MTD according to hitherto data. Simultaneously, EWOC prohibits dose escalation to levels with a posterior probability > 33.3% for a DLT rate > 0.35. Since formal sample size calculations are not feasible for phase I dose escalation trials, the following stopping criteria were established instead:

- The MTD can be estimated with sufficient certainty (dose level with a posterior probability of a DLT rate in the target interval > 75%)
- Four consecutive dose recommendations are for the same dose level
- All dose levels are deemed too toxic (two consecutive complete overdose scenarios according to EWOC)
- A time limit of 40 months is reached (recruitment of current dose escalation cohort may be completed)
- A patient limit of 36 patients is reached (recruitment of current dose escalation cohort may be completed, yielding a patient maximum of 38)

After stopping patient accrual and completion of individual observation time in all patients, a final dose recommendation will be calculated, which represents the MTD estimate. The characteristics of the trial design, including correct MTD estimation, overdosing rate and sample size, were assessed in simulations of 10.000 trials for each of four different dose-toxicity scenarios (conservative, low toxicity, early excessive toxicity, late excessive toxicity).

### Follow-up

Patients are followed-up 6–8 weeks after SBRT and then three-monthly for at least 24 months. Visits include a clinic assessment as well as a thoracic CT scan. Toxicity will be documented according to the CTCAE in version 5.0. The translational program encompasses measurement of cytokine levels and immunophenotyping of PBMC (before treatment, then 3-mothly for the first year) as well as multiparametric thoracic MRI (before treatment and 3 months after treatment).

## Discussion

SBRT of ULT remains a clinical challenge, where the risk for severe toxicity must be weighed against a potentially compromised local tumor control. Currently, the Canadian SUNSET trial investigates dose escalated SBRT of ULT using non-adaptive CT-based treatment techniques [14]. Compared to MAGELLAN, definition of ultracentral location is wider and encompasses PTV overlap with the PBT, esophagus and great vessels. Recent reports suggest that toxicity following SBRT of ULT is mainly associated with dose to the PBT [4, 6], whereas the esophagus is the most radiosensitive mediastinal OAR [15]. Accordingly, the MAGELLAN trial will focus on treatment of the very high-risk tumors in proximity to the PBT and esophagus, thereby exploiting the potential of MR-guidance to correct for inter- as well as intrafractional anatomical changes. Since an analysis of our institutional database demonstrated that application of 10 × 5 Gy to ULT is safe using CT-based standard techniques [7], 10 × 5 Gy was chosen as de-escalation level 0 and dose escalation starts at 10 × 5.5 Gy. The highest dose level is 10 × 6.5 Gy, which should yield a favorable local tumor control because it confidently reaches an ablative BED_10_ (with an α/β ratio = 10 for tumor cells) > 100 Gy [2]. Further dose escalation might not significantly improve local control, but risk disproportionate complications. This also means that the MAGELLAN might not reach the true MTD if the highest dose level yields a favorable DLT rate. Instead, it would just escalate to an effective dose. To strengthen patient safety, OAR constraints were developed according to the best available evidence [15, 16]. In contrast to the HILUS trial, which allowed a dose maximum of approximately 150% inside the PTV [6], we will restrict the maximum PTV dose to 125% to avoid potentially dangerous dose hot spots close to the PBT. Furthermore, a TITE-CRM design with EWOC modification is applied. The additional backup element is an innovative development of TITE-CRM, which allows continuous patient accrual and treatment as close to the assumed MTD as safely possible. In the future, the results of the MAGELLAN trial will pave the way for further prospective investigations and guide the clinical implementations of MR-guided SBRT for ablative treatment of ULT.

## Data Availability

Not applicable.
